# Deep Learning-Based Protein Features Predict Overall Survival and Chemotherapy Benefit in Gastric Cancer

**DOI:** 10.3389/fonc.2022.847706

**Published:** 2022-05-16

**Authors:** Xuefei Zhao, Xia Xia, Xinyue Wang, Mingze Bai, Dongdong Zhan, Kunxian Shu

**Affiliations:** ^1^ Chongqing Key Laboratory of Big Data for Bio Intelligence, School of Bioinformation, Chongqing University of Posts and Telecommunications, Chongqing, China; ^2^ State Key Laboratory of Proteomics, Beijing Proteome Research Center, National Center for Protein Sciences (Beijing), Beijing Institute of Lifeomics, Beijing, China; ^3^ Department of Bioinformatics, Beijing Pineal Diagnostics Co., Ltd., Beijing, China

**Keywords:** proteomics, gastric cancer, deep learning, autoencoder, molecular subtyping, chemotherapy benefit

## Abstract

Gastric cancer (GC) is one of the most common malignant tumors with a high mortality rate worldwide and lacks effective methods for prognosis prediction. Postoperative adjuvant chemotherapy is the first-line treatment for advanced gastric cancer, but only a subgroup of patients benefits from it. Here, we used 833 formalin-fixed, paraffin-embedded resected tumor samples from patients with TNM stage II/III GC and established a proteomic subtyping workflow using 100 deep-learned features. Two proteomic subtypes (S-I and S-II) with overall survival differences were identified. S-I has a better survival rate and is sensitive to chemotherapy. Patients in the S-I who received adjuvant chemotherapy had a significant improvement in the 5-year overall survival rate compared with patients who received surgery alone (65.3% vs 52.6%; log-rank P = 0.014), but no improvement was observed in the S-II (54% vs 51%; log-rank P = 0.96). These results were verified in an independent validation set. Furthermore, we also evaluated the superiority and scalability of the deep learning-based workflow in cancer molecular subtyping, exhibiting its great utility and potential in prognosis prediction and therapeutic decision-making.

## Introduction

Gastric cancer (GC) is one of the most common malignant tumors in humans and is the fourth leading cause of cancer death in the world, especially in Asia (1). According to the World Health Organization (WHO) statistics, the global morbidity of GC in 2020 was 6.6%, and mortality was 7.7% (2), making it an important global health issue (3, 4).

The high morbidity and mortality rates of GC reflect the insufficiency of diagnosis and treatment. Although immunotherapy has been approved for first-line treatment of GC, only a small percentage of patients benefit from it. Trastuzumab remains the only approved first-line therapy for HER2-positive GC (5–8), but the HER2-positive rate for GC is only 10.4 to 20.2% globally (9). Chemotherapy is still the main treatment for HER2-negative GC patients (10–15). However, a GC phase II clinical trial reported that about 60% of patients responded to chemotherapy, most patients developed drug resistance within a few months (16). The overall benefit of chemotherapy in GC is limited (10, 17–19). Therefore, it is crucial to identify the chemotherapy benefit groups for advanced HER2-negative GC patients.

With the advancement of omics technology, recent studies have focused on molecular subtyping while considering the conventional pathological classification. For example, the molecular subtyping of GC has provided an opportunity for individualized treatment (20–23). The Cancer Genome Atlas (TCGA) proposed four GC molecular subtypes: chromosomal instability (CIN), microsatellite instability (MSI), genome stability (GS), and Epstein–Barr virus (EBV) positivity. Of these, EBV and MSI might benefit from immunotherapy, while CIN and GS were less likely to respond to immunotherapy. These results indicate that molecular subtyping can guide immunotherapy. Likewise, the Asian Cancer Research Group (ACRG) has also defined four GC molecular subtypes based on the epithelial-to-mesenchymal transition (EMT), microsatellite instability (MSI), and TP53 activity: MSI, microsatellite stable (MSS)/EMT, MSS/TP53^+^ and MSS/TP53^−^ (24, 25). These subtypes have different survival outcomes, suggesting that molecular subtyping can imply prognosis. While clearly representing milestones in the field, these studies did not reveal the relationship between GC subtypes and chemotherapy. More recent molecular subtyping studies have indeed established a correlation with the clinical characteristics (26–28). For example, Oh et al. identified two subtypes based on genomic data of GC: mesenchymal phenotype (MP) and epithelial phenotype (EP), which are linked to distinct patterns of molecular alterations, disease progression, and prognosis (29). The MP subtype was associated with poor prognosis and resistance to chemotherapy, while the EP subtype was associated with good prognosis and benefit from chemotherapy. Due to the limited number of patients receiving chemotherapy, the relationship between subtypes and chemotherapy has not been verified in independent cohorts. These studies indicated that molecular subtyping can identify which GC patients are most likely to benefit from adjuvant chemotherapy.

Recently, Ge et al. analyzed the proteomic of diffuse-type gastric cancer (DGC) with 84 pairs of tumors and their nearby tissues and obtained three molecular subtypes: cell cycle (PX1), EMT (PX2), and immunological process enrichment subtype (PX3) (30). These subtypes are strongly associated with survival outcomes and chemotherapy sensitivity. However, due to the limited amount of data, this result needs to be further verified. In a subsequent proteomic subtyping of GC, a workflow based on non-negative matrix factorization (NMF) consensus clustering was applied on 1,020 formalin-fixed, paraffin-embedded (FFPE) GC samples (31). While this workflow could identify chemotherapy benefit for patients, there was no significant difference in prognosis between the two molecular subtypes.

Recently, deep learning (DL) has gained increasing attraction and has been widely applied in various aspects of biological research (32, 33), namely, in biomedicine (34), clinical diagnosis (35), bioinformatics (36), and other life science related fields (37, 38). For example, a preoperative computed tomography (CT) image-based signature constructed by a deep neural network can predict overall survival (OS) and chemotherapy benefit in GC (39). A study based on breast cancer genomic data described how a single nonlinear hidden node extracted by an autoencoder (AE) framework can characterize survival differences (40). Moreover, a recent study using an AE framework combined with multi-omics data to extract nonlinear features from hepatocellular carcinoma can discover survival-sensitive molecular subtypes (41). These achievements suggest the potential for DL in GC prognosis studies with proteomics data.

In this study, we developed a DL-based workflow that embeds the AE framework and applied it to the proteomic profile collected on resected FFPE tumor samples from 833 patients with TNM stage II/III GC. Patients were classified into two subgroups (S-I and S-II) with OS differences. S-I has a better survival rate and is sensitive to chemotherapy. Moreover, we compared the prognostic predictive ability of the features extracted from AE with two alternative methods. Finally, we further test the scalability of the workflow in two external validation sets.

## Materials and Methods

### Study Design and Patient Cohorts

In this study, we used FFPE surgical resection samples from 833 GC patients with TNM stage II/III from previous work (31). These samples were collected between 2004 and 2016 and came from five hospitals, namely, the Peking University Cancer Hospital & Institute (PKUCH, N = 387), the Fourth Medical Center of PLA General Hospital/304 Hospital (304H, N = 210), the Xijing Hospital of Digestive Diseases (XJH, N = 112), the Medical School of Chinese PLA/301 Hospital (301H, N = 71), and the Shanxi Cancer Hospital (SXCH, N = 53). Patients had provided written informed consent and had complete follow-up records with adequate clinical annotations. The median follow-up time was 3.7 years (a range of 0.08 to 10.4 years).

We used the data in three steps: The first step is to split the data into a discovery set (PKUCH and XJH) and an independent validation set (304H, 301H, and SXCH). The features extracted from the whole discovery set by AE were used for consensus clustering to obtain labels of survival-risk subtypes. The second step is to train the classifier model by dividing the discovery set into training and test sets at a ratio of 7:3. The third step uses data from an independent validation set to evaluate the prediction accuracy of the DL-based prognosis model.

### Features Extraction Using a DL Framework

We use proteomic data from 499 samples from the discovery set as the input for the AE framework to feature transformation. AE consists of an encoder and a decoder (42), which is a feed-forward and non-recursive neural network commonly used in semi-supervised and unsupervised learning (33, 43). Given an input layer with an input x = (x_1_, x_2_, …… x_n_) of dimension n, the objective of an AE is to reconstruct x with the output x’ (x and x’ have the same dimension) *via* transforming x through successive hidden layers.

For the hidden network layer, we use *Relu* as the activation function between the input layer x and the output layer y. That is:


y=f(x)=max (0,x)

We use *Sigmoid* as the activation function for the reconstructed layer. That is:


$$y=f(x)=\frac{1}{1+e^{-x}}$$

However, the bottleneck layer does not use any activation functions.

The objective of AE training is to find the different weight vectors W_i_, minimizing a specific objective function. We chose mean-square error *(MSE)* as the objective function, which measures the error between the input x and the output x’.


$$\text{MSE}(\text{x},{\text{x}}^{\prime })=\frac{1}{n}\sum_{i=1}^{n}\left(x_{i}-{x^{\prime }}_{i}\right){}^{2}$$

Here, n refers to the sample number, x_i_ and x_i_’ refer to the input and output values of the current sample, respectively.

We constructed the AE with three hidden layers (500, 100, and 500 nodes, respectively) using Python’s Keras library (https://github.com/fchollet/keras). The bottleneck layer of the AE was used to generate novel features. Finally, the AE was trained with the *Adam* optimizer as the optimization function and 0.001 as the learning rate. A gradient descent algorithm with 80 epochs was used. Epoch here refers to the iteration of the learning algorithm on the whole training data set.

### Consensus Clustering

The AE reduced the original features to 100 new features obtained from the bottleneck layer. For these transformation features generated by AE, we use the R package ConsensusClusterPlus (44) to perform consensus clustering. We determined the optimal number of clusters with two metrics: (1) Silhouette index and (2) log-rank P-value. The clustering algorithm was k-means using Euclidean distance. The proportion of samples selected was 80% in each resampling, and the number of clusters considered was 2 to 5. Among them, a consensus matrix with k = 2 appeared to have the clearest cut between clusters and showed a significant association with the survival of the patients.

### Supervised Classification

After obtaining the labels through consensus clustering, we built three supervised classification models, namely, random forest (RF), logistic regression (LR), and support vector machines (SVM). Using the univariate Cox proportional hazards (Cox-PH) model, we identified 56 prognosis-related proteins (log-rank P <0.01) used for training classifiers.

The expression values of these 56 proteins on the training and test sets first standardized with Z-Score before being entered into classifiers. The average areas under the curve (AUC) of models were evaluated on the training set combined with 10-fold cross-validation (CV), and then these models were further determined based on their performance on the test set.

We built three classifiers using Python’s scikit-learn package that could be used for performing grid search to find the best hyperparameters of the three models. Finally, a RF model containing 10 trees was determined.

### Alternative Approaches to the AE Framework

**Figure 1:**
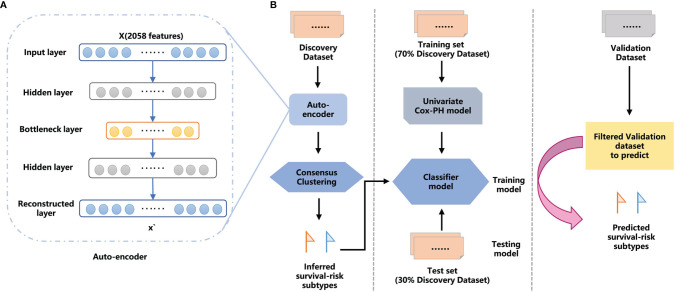
The overall workflow for GC proteomic molecular subtyping based on DL AE framework features extraction. **(A)** AE framework. **(B)** Workflow combining DL and machine learning (ML) methods to predict GC survival-risk subtypes. The workflow includes two steps: step 1, inferring survival-risk subtypes in the discovery set; and step 2, predicting survival-risk labels for samples in the independent validation set.

To verify the advantages of features transformed from AE, we compared the performance of AE with traditional machine learning methods. Here, Principal Component Analysis (PCA), a traditional linear dimension reduction method, and Uniform Manifold Approximation and Projection (UMAP) (45), a new nonlinear dimension reduction algorithm, were used to find the optimal number of retained features, respectively. The samples were then clustered using the same consensus clustering procedure (**Figure 1**).

### Differential Expression Analysis

A differential expression analysis was performed to identify the differentially expressed proteins between the two survival subtypes. The Mann-Whitney U test was used to identify proteins with significantly different expression between the two subtypes, and the Benjamini–Hochberg method was used to adjust the P-values.

### Enriched Pathway Analysis

We used the Reactome pathway database (https://reactome.org/) (46) to perform functional enrichment analysis for the differentially expressed proteins of the two subtypes.

## Results

### Study Design and Clinical Characteristics

A total of 833 FFPE GC samples from five hospitals were used in this study. There were 309 (37%) TNM stage II patients and 524 (63%) TNM stage III patients, 582 (70%) of whom received adjuvant chemotherapy.

The median of protein detection in the five hospitals was between 1,273 and 1,543 (**Supplementary Figure 1A**). Moreover, the PCA showed no clear boundaries in the five hospitals, suggesting that there was no batch effect caused by the source of samples (**Supplementary Figure 1B**). We combined two hospitals as discovery sets and randomly divided them into the training and test sets at a ratio of 7:3. The remaining three hospitals were combined as an independent validation set. As shown in **Supplementary Figures 1C, D**, the sample distribution of the three data sets is relatively balanced. **Table 1** lists detailed clinicopathological information for patients in the training set (n = 349), the test set (n = 150), and the independent validation set n = 334.

**Table 1:** Clinical information of patients in this study.

	Discovery dataset		Validation dataset
	Training set	Test set	
**Patients**			
Total	349	150	334
Phase II	129	53	127
Phase III	220	97	207
**Age (year)**			
Mean ± SD	59.6 ± 12.1	59.3 ± 12.2	61.6 ± 11.1
Median	60.0	60.0	62.0
Range	25–87	20–81	27–89
**Gender**			
Male	257	106	270
Female	92	44	64
**Chemotherapy**			
Yes	249	104	229
No	73	35	95
**Overall Survival**			
Death	168	71	142
Live	181	79	192

We established a workflow based on the AE framework, with the structure highlighted in **Figure 1A**. We used the 100 nodes from the bottleneck-hidden layer of AE as new features, and then conducted consensus clustering for the hidden features. The silhouette index and log-rank P-value were used to judge the quality of clustering and to obtain the optimal number of clusters on the discovery set. Next, we used a univariate Cox-PH model to obtain prognosis-related proteins, which were used as features to establish a classifier. We trained the classifier with a 10-fold CV in the training set and tested it in the test set, then further validated it in an independent validation set. The workflow is shown in **Figure 1B**.

### Two Subtypes With Differential Overall Survival Were Identified in the Discovery Set

**Figure 2:**
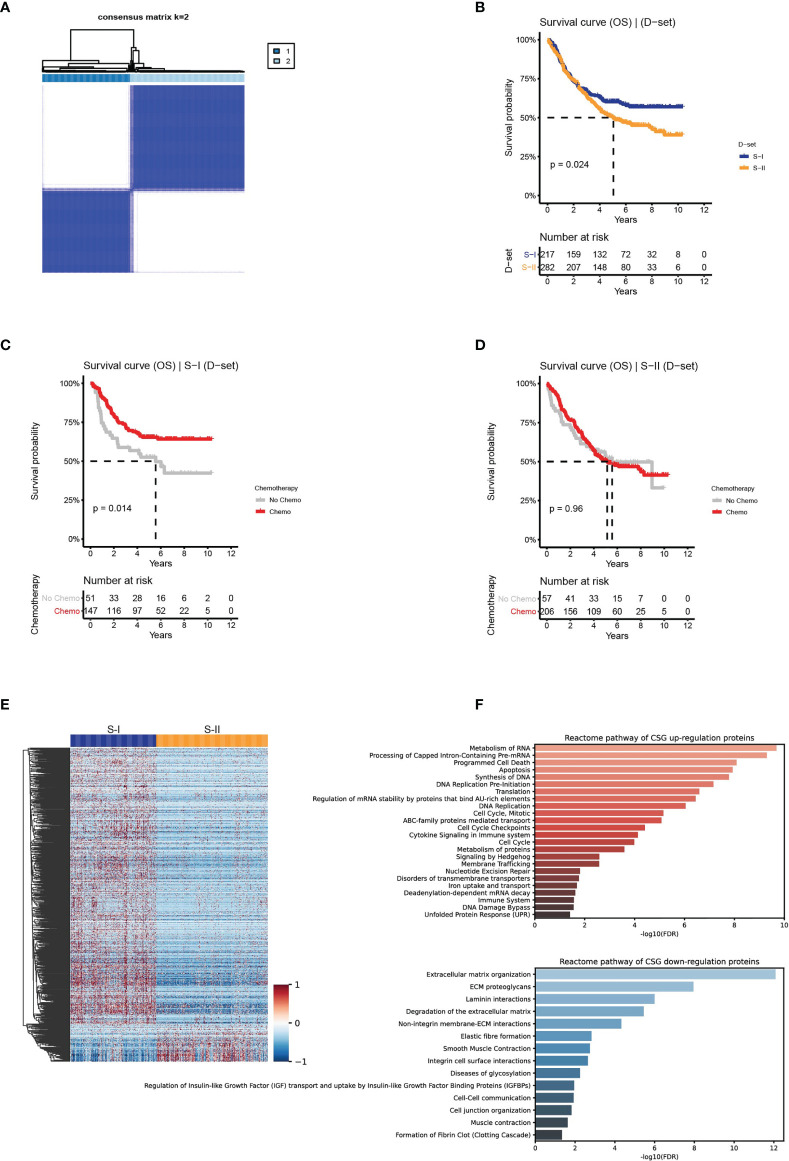
Clinical outcomes and differentially expressed proteins with their enriched pathways of the molecular subtypes in the discovery set. **(A)** The discovery set was clustered using the ConsensusClusterPlus method based on the protein features transformed from AE. **(B)** The OS of S-I and S-II. **(C, D)** The OS by chemotherapy status for S-I and S-II. **(E)** Differentially expressed proteins in the two subtypes. **(F)** Reactome revealed the pathways that were significantly enriched in the proteomic subtypes.

For the discovery set, we retained proteins detected in one-tenth (n = 49) of all samples, resulting in 2,058 proteins that were used for further analysis. These 2,058 proteins were transformed by the AE, and 100 nonlinear features were retained for consensus clustering. When K = 2, the consensus matrix exhibited the clearest cut among clusters (**Figure 2A**), with an average silhouette index of 0.97 (**Supplementary Figure 2A**).

The association between prognosis and chemotherapy within each subtype was analyzed. Significant differences in OS were found between the two subtypes: S-I with good prognosis (n = 217, 43%) and S-II with poor prognosis (n = 282, 57%) (log-rank P = 0.024, **Figure 2B**). Additionally, we found that the 5-year OS rate of the S-I patients who received adjuvant chemotherapy was 65.3%, which is a significantly increased 12% compared with 52.6% for patients who received surgery only (**Figure 2C**). While no significant differences were observed in the 5-year OS rate for S-II between patients who received adjuvant chemotherapy (54%) and those who did not receive chemotherapy (51%) (**Figure 2D**).

We adopted the Mann–Whitney U test to perform differential expression analysis in two subtypes. Of the 884 differentially expressed proteins obtained (FDR <0.05 & fold change >2), 783 proteins were upregulated in S-I, and 101 proteins were upregulated in S-II (**Figure 2E** and **Supplementary Figure 2B**). Using the differentially expressed proteins above, we performed pathway enrichment analysis in the Reactome pathway database (46) to determine the pathways enriched in the two subtypes. S-I showed the characteristics of cell proliferation, mainly enriched in DNA replication, cell cycle, and programmed cell death. S-II showed the characteristics of the tumor microenvironment (TME), which was mainly enriched in the extracellular matrix (ECM)-related pathways (**Figure 2F**).

### The Overall Survival Subtypes were Validated in an Independent Validation Set

**Figure 3:**
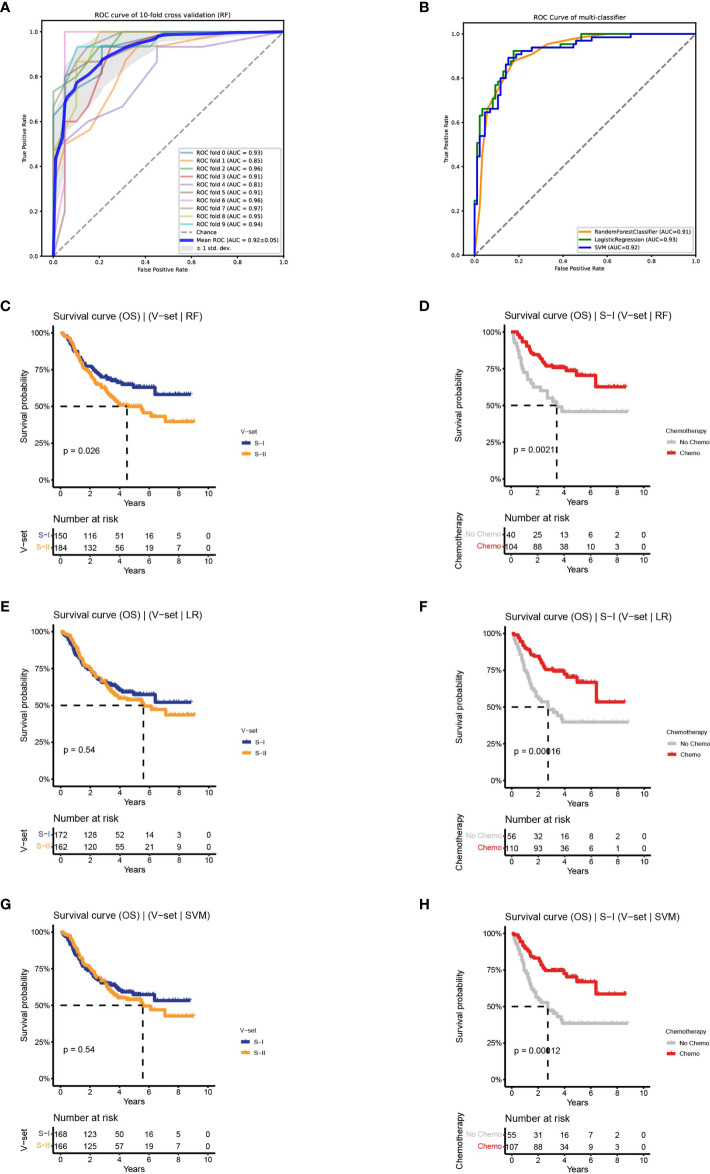
Classifiers were established to predict survival-risk labels for samples in the independent set. **(A)** The receiver operating characteristic (ROC) curve of RF on training set with 10-fold CV. **(B)** The ROC curve of three classifiers on test set. **(C, D)** The Kaplan–Meier (K–M) curves of the subtypes in independent set predict by RF: OS (left) and OS by chemotherapy status of S-I (right). **(E, F)** The K–M curves of the subtypes in independent set predict by LR: OS (left) and OS by chemotherapy status of S-I (right). **(G, H)** The K–M curves of the subtypes in independent set predict by SVM: OS (left) and OS by chemotherapy status of S-I (right).

To evaluate the prognostic prediction accuracy of the DL-based workflow, we established a classifier using the two subtypes identified above as labels. Using a Cox-PH model, we obtained 56 prognosis-related proteins as features to train the classifier. The heat map of 56 proteins is shown in **Supplementary Figure 3A**. To build the classifier, we evaluated three commonly used machine learning (ML) models, namely, RF, LR, and SVM. The three models resulted in an average AUC of 0.92, 0.91, and 0.89 on the training set with a 10-fold CV, and 0.91, 0.93, and 0.92 on the test set, respectively (**Figures 3A, B** and **Supplementary Figures 3B, C**).

Based on the performance on the training and test sets, we observed that the three ML models performed equally well. To determine the robustness of the classifiers in predicting OS outcomes, we applied the three models to an independent validation set containing 334 patients. Subtypes of the validation set were predicted by the three models, followed by an association analysis between prognosis and chemotherapy within each subgroup. We discovered that there was a difference in OS between the two subtypes predicted by RF on the validation set (log-rank P = 0.026). Among the predicted S-I with good prognosis (n = 150, 45%), the 5-year OS rate of patients receiving adjuvant chemotherapy was increased by 25% compared to patients receiving surgery alone (70.5% vs 45.8%), consistent with the characteristics of the S-I in the discovery set. The predicted S-II with poor prognosis (n = 184, 55%) also showed similar characteristics as the S-II in the discovery set, and there was no significant difference in the 5-year OS rate between the chemotherapy group and the non-chemotherapy group (50% vs 46%, log-rank P = 0.026) (**Figures 3C, D** and **Supplementary Figure 3D**). In contrast, LR and SVM can only predict chemotherapy benefit, but not prognosis (**Figures 3E–H** **and** **Supplementary Figures 3E, F**). Collectively, the results show that the two subtypes were verified on the independent validation set through the RF classifier.

### The DL-Based Methodology Outperforms Two Alternative Approaches

To verify the advantages of features transformed from AE in predicting prognosis and chemotherapy benefit, we compared them with two alternative approaches: PCA and UMAP (45).

**Figure 4:**
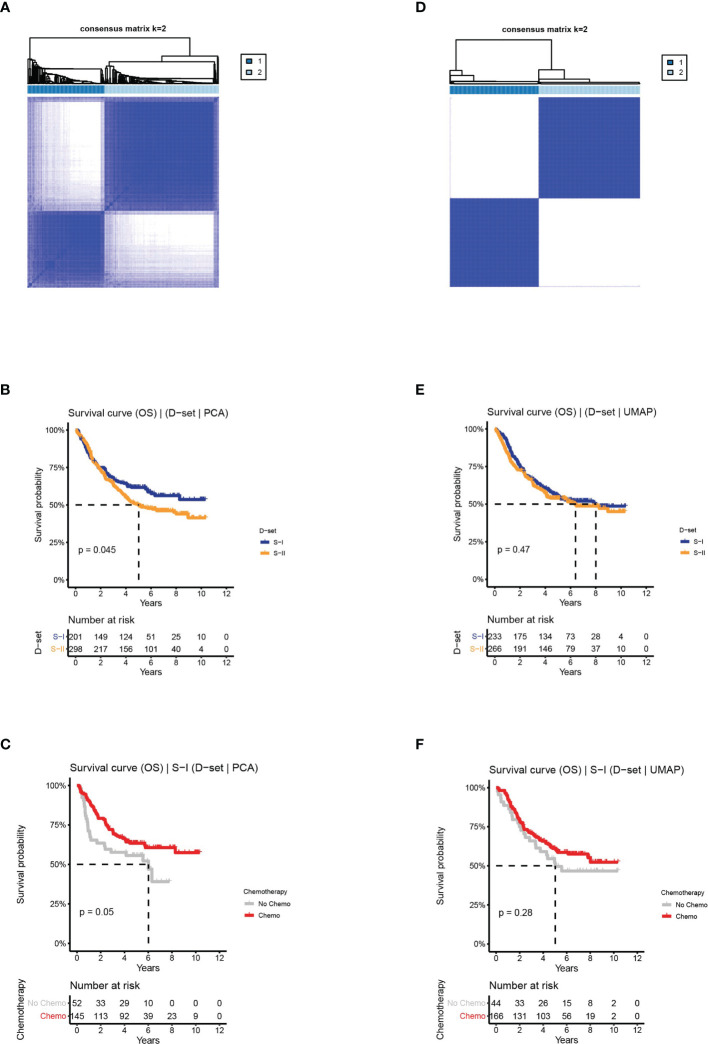
The clinical outcomes of molecular subtypes obtained from two alternative approaches in the discovery set. **(A)** The clustering results, **(B)** the OS of S-I and S-II, and **(C)** the OS by chemotherapy status for S-I, obtained from PCA. **(D)** The clustering results, **(E)** the OS of S-I and S-II, and **(F)** the OS by chemotherapy status for S-I, obtained from UMAP.

In the first approach, we reserved the optimal 44 principal components for consensus clustering. Although the consensus matrix did not have a clear boundary (**Figure 4A**), this approach could detect survival subtypes with a significant log-rank p value (log-rank P = 0.045, **Figure 4B**). Additionally, the two subtypes also exhibited similar characteristics in terms of prognosis and chemotherapy benefit with S-I (log-rank P = 0.05, **Figure 4C**) and S-II (log-rank P = 0.66, **Supplementary Figure 4A**). However, compared with the clustering results obtained from the AE, the silhouette index of clustering obtained from PCA is only 0.73 (**Supplementary Figure 4B**), with less significant survival differences.

In the second approach, we used optimal 90 features extracted from UMAP for consensus clustering, obtaining two subtypes with a silhouette index of 0.99. However, there was no difference in OS or chemosensitivity (log-rank P = 0.47, S-I: log-rank P = 0.28, S-II: log-rank P = 0.28, **Figures 4D–F** and **Supplementary Figures 4C, D**).

Compared with PCA and UMAP, hidden features extracted by AE could better distinguish the OS differences between S-I and S-II (**Table 2**). Further, we found that when the number of hidden layers was greater than three, the learning ability of the AE was decreased. However, with only one hidden layer, the features extracted by AE do not have the ability to predict prognosis. When only three hidden layers were set, too few nodes of hidden layers could also lead to the decline of network learning ability. Therefore, when the network with three hidden layers and learning ability was similar, we still chose the network with relatively few nodes (**Table 3**).

**Table 2:** Performance of AE and two alternative approaches.

Method	Components (n)	Average Silhouette Index	P-value (S-I vs S-II)	P-value (S-I)	P-value (S-II)
AE	100	0.97	0.024	0.014	0.96
PCA	30	0.69	0.69	0.21	0.48
	44	0.73	0.045	0.05	0.66
	50	0.66	0.032	0.14	0.39
	60	0.72	0.27	0.051	0.73
UMAP	30	0.87	0.32	0.36	0.2
	60	0.97	0.45	0.31	0.24
	90	0.99	0.47	0.28	0.28
	120	0.94	0.98	0.25	0.29

**Table 3:** Performance of AE with different hidden layers and nodes in discovery set.

Hidden layers (N)	Nodes (N)	Average Silhouette Index	P-value (S-I vs S-II)	P-value (S-I)	P-value (S-II)
1	100	0.98	0.99	0.033	0.47
1	500	0.98	0.13	0.034	0.5
1	1,000	0.97	0.13	0.034	0.5
3	100, 50, 100	0.64	0.027	0.017	0.89
3	500, 100, 500	0.97	0.024	0.014	0.96
3	1,500, 500, 1,500	0.95	0.026	0.015	0.82
5	500, 100, 50, 100, 500	0.87	0.0096	0.05	0.51
5	1,000, 500, 100, 500, 1,000	0.96	0.024	0.0086	0.69
5	2,000, 1,200, 500, 1,200, 2,000	0.95	0.057	0.048	0.65

### The Scalability of the Workflow Was Verified in External Validation Sets

To test the scalability of the DL-based GC subtyping workflow, we used two sets of public clinical GC data for verification, namely, proteome data obtained from frozen tissues of 75 TNM stage II/III DGC patients (30) and RNA-seq data of 247 TNM stage II/III GC patients (47). Of the 247 GC patients, 124 were treated with Uracil-Tegafur (UFT) and 123 were treated with a combination of paclitaxel and UFT (PacUFT), and all received chemotherapy.

**Figure 5:**
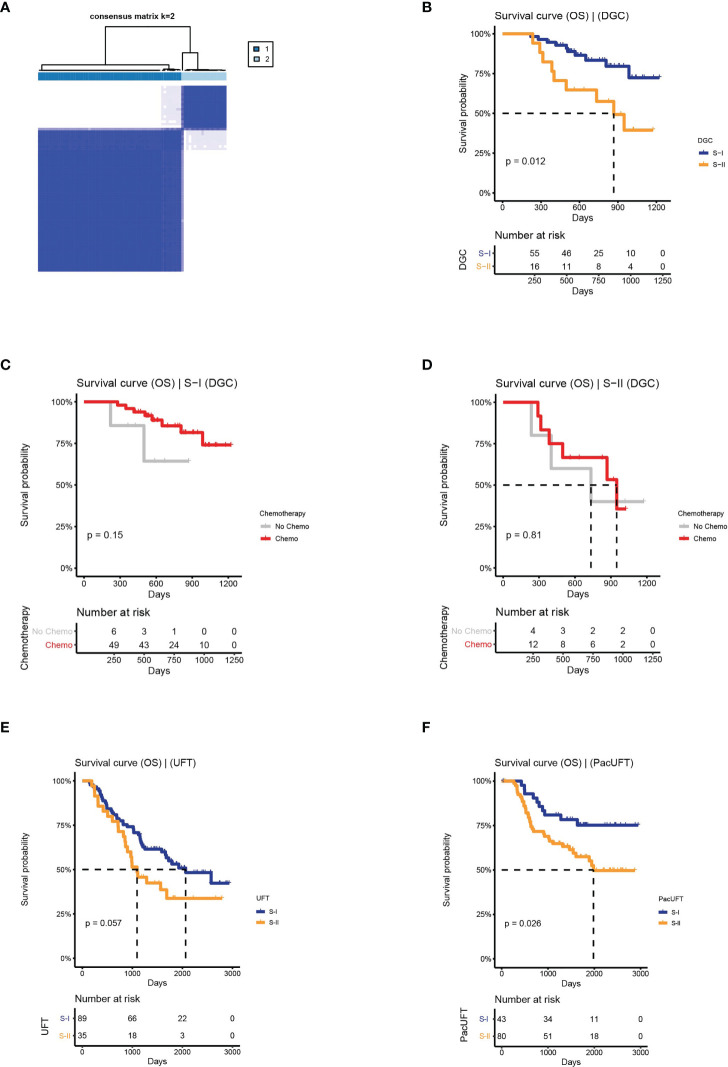
The clinical outcomes of molecular subtypes obtained by the protein features transformed from AE in the external validation set. **(A)** The clustering result of DGC. **(B)** The OS of S-I and S-II. **(C, D)** The OS by chemotherapy status for S-I and S-II. **(E)** The OS of molecular subtypes for UFT-treated and. **(F)** PacUFT-treated patients in the external validation set.

Based on the above workflow, we found that the prognosis of frozen samples of DGC could also be distinguished (log-rank P = 0.012, **Figures 5A, B**). The subtype with a good prognosis exhibited a chemo-benefit trend, which was consistent with that of the S-I identified in FFPE samples, although the p-value of the S-I was not significant (log-rank P = 0.15, **Figure 5C**), likely due to the limited data. Whereas the subtype with poor prognosis showed the same characteristics as the S-II identified in FFPE samples (log-rank P = 0.81, **Figure 5D**). This indicates that our deep learning-based GC subtyping workflow not only has good prognosis prediction and screening ability for chemotherapy benefit in FFPE but can also be applied to frozen tissues.

Similarly, our workflow was used to classify 124 GC patients treated with UFT and 123 GC patients treated with PacUFT, respectively. We obtained two subtypes with OS differences from each of the two groups (UFT: log-rank P = 0.057; PacUFT: log-rank P = 0.026; **Figures 5E, F**). Among them, patients with a subtype with a better prognosis benefited from chemotherapy, whereas patients with a subtype with a poor prognosis resisted chemotherapy.

These results demonstrated that our DL-based GC subtyping workflow was scalable to some extent, and that it could predict prognosis and screen for the chemotherapy benefit on GC samples from various sources such as proteomes and transcriptomes. This workflow may provide a new clinically applicable strategy for determining which patients are more likely to benefit from adjuvant chemotherapy.

## Discussion

Accurate prediction of prognosis and treatment response is crucial for risk stratification and management of cancer patients (39). In this study, we established a workflow for molecular subtyping of GC based on AE framework feature extraction. This workflow, which could not only predict the OS outcomes for GC patients but also identify the chemotherapy benefit, was validated on two independent clinical GC datasets.

The diagnosis and treatment of GC have been advanced over the past few decades, but most GC patients are still diagnosed at an advanced stage (48) and the targeted therapies are not sufficient. For HER2-negative advanced GC patients, the primary treatment is still limited to platinum, fluoropyrimidine, and paclitaxel chemotherapeutic drugs (15). Furthermore, there are significant individual differences in prognosis even among patients at the same stage receiving the same treatment (10, 18, 49–51). Some patients clearly benefit from chemotherapy, while some patients do not and may have a worse prognosis due to the toxic effects of chemotherapy (10, 16, 17). Since the overall benefit of adjuvant chemotherapy for GC is limited (17–19), predicting which specific patients will benefit from chemotherapy is critical. Studies have been conducted on the benefits of chemotherapy for GC (10, 52, 53), but relevant work is still lacking. Therefore, it is urgent to find biomarkers or features to better predict prognosis and guide treatment strategies.

We conducted a retrospective proteomic analysis of 833 clinically-ready FFPE GC samples from 5 independent centers. In this study, we proposed a DL-based proteomic subtyping workflow to predict the prognosis of GC patients with stage II/III and chemotherapy benefit. We found that approximately 43% of patients in the discovery set benefited from adjuvant chemotherapy, and this group had a better prognosis than those who did not benefit from chemotherapy. Pathway enrichment analysis of the S-I and S-II showed that they had different active pathways. S-I exhibited the characteristics of cell proliferation, while S-II was a TME. The S-II was mainly enriched in ECM-related pathways. Interestingly, it has been reported in relevant studies that ECM can form a physical barrier to anticancer drugs (54, 55) and prevent the effects of chemotherapy and immunotherapy, so the deposition of ECM is associated with poor prognosis of various tumors (49). One of the representative ECM genes, *FBN1*, is up-regulated in S-II. Relevant studies have verified that knocking out this gene can make cancer cells sensitive to chemotherapy drugs (49). Therefore, determining the characteristics of the ECM microenvironment in patients with chemotherapy insensitivity can help predict prognosis and chemotherapy response and provide indications for treatment.

Additionally, we proved the superiority of using AE over PCA and UMAP to extract features and perform consensus clustering in predicting prognosis and chemotherapy benefit. The superiority may result from the ability of AE to capture complex relationships between analytes through multi-layer neural network transformation.

Despite this, it has several limitations in this study. First, this is a retrospective study, and these results need to be verified in future randomized clinical trials. Second, there was a lack of further in-depth examination of chemotherapy benefits or chemotherapy resistance mechanisms for the two subtypes.

In conclusion, we established and verified a workflow for GC proteomic molecular subtyping based on features extracted from AE, which can provide prognostic value for GC patients and distinguish chemotherapy benefit groups. Additionally, we also demonstrated the superiority and scalability of the DL-based workflow in cancer molecular subtyping, exhibiting its great application potential in therapeutic decision making and prognosis prediction. Further validation of these findings in a multicenter prospective study is warranted.

## Data Availability Statement

The MS raw data generated in this study have been submitted to ProteomeXchange database (www.proteomexchange.org) via the iProx partner repository (56) under accession number IPX0004364001.

## Author Contributions

XZ designed this study. XZ and XX performed the data analysis and prepared the figures. XW wrote the manuscript. MB revised the content. DZ and KS were responsible for confirming the authenticity of the data. All authors listed have made a substantial, direct, and intellectual contribution to the work and approved it for publication.

## Funding

The current study was funded by the National Key Research and Development Program of China (2018YFA0507504), National Natural Science Foundation of China (Grant No. 31971360).

## Conflict of Interest

Author DZ was employed by Beijing Pineal Diagnostics Co., Ltd.

The remaining authors declare that the research was conducted in the absence of any commercial or financial relationships that could be construed as a potential conflict of interest.

## Publisher’s Note

All claims expressed in this article are solely those of the authors and do not necessarily represent those of their affiliated organizations, or those of the publisher, the editors and the reviewers. Any product that may be evaluated in this article, or claim that may be made by its manufacturer, is not guaranteed or endorsed by the publisher.

## Supplementary Material

Supplementary Figure 1 Data quality evaluation. **(A)** Box plot of protein identification numbers in five hospitals. **(B)** PCA of 833 samples in five hospitals. **(C)** Violin plot of protein identification numbers in three datasets. **(D)** Box plot of protein abundance in each sample in three datasets.

Supplementary Figure 2 The clustering result and differentially expressed proteins of the molecular subtypes in discovery set. **(A)** The silhouette plot of clustering results obtained from AE. **(B)** Volcano plot of differentially expressed proteins in two subtypes.

Supplementary Figure 3 Classifiers were established to predict survival-risk labels for samples in independent set. **(A)** A heat map based on the 56 selected proteins expression. **(B, C)** The ROC curve of LR and SVM on training set with 10-fold CV, respectively. **(D–F)** The OS by chemotherapy status for S-II predict by RF, LR, and SVM, respectively.

Supplementary Figure 4 The clustering results obtained from two alternative approaches in discovery set. **(A)** The silhouette plot of clustering results and **(B)** the OS by chemotherapy status for S-II obtained from PCA. **(C)** The silhouette plot of clustering results and **(D)** the OS by chemotherapy status for S-II obtained from UMAP.

Supplementary Figure 5 The clustering results obtained by the protein features transformed from AE in the external validation set. **(A)** The silhouette plot of clustering results in DGC. **(B, C)** The clustering results for UFT-treated and **(D, E)** PacUFT-treated patients in the external validation set.
