# Supplementary figures and images for: Deep Learning-Based Protein Features Predict Overall Survival and Chemotherapy Benefit in Gastric Cancer
Source: Front Oncol. 2022 May 16;12:847706. doi: 10.3389/fonc.2022.847706 (PMC9148960; doi:10.3389/fonc.2022.847706)

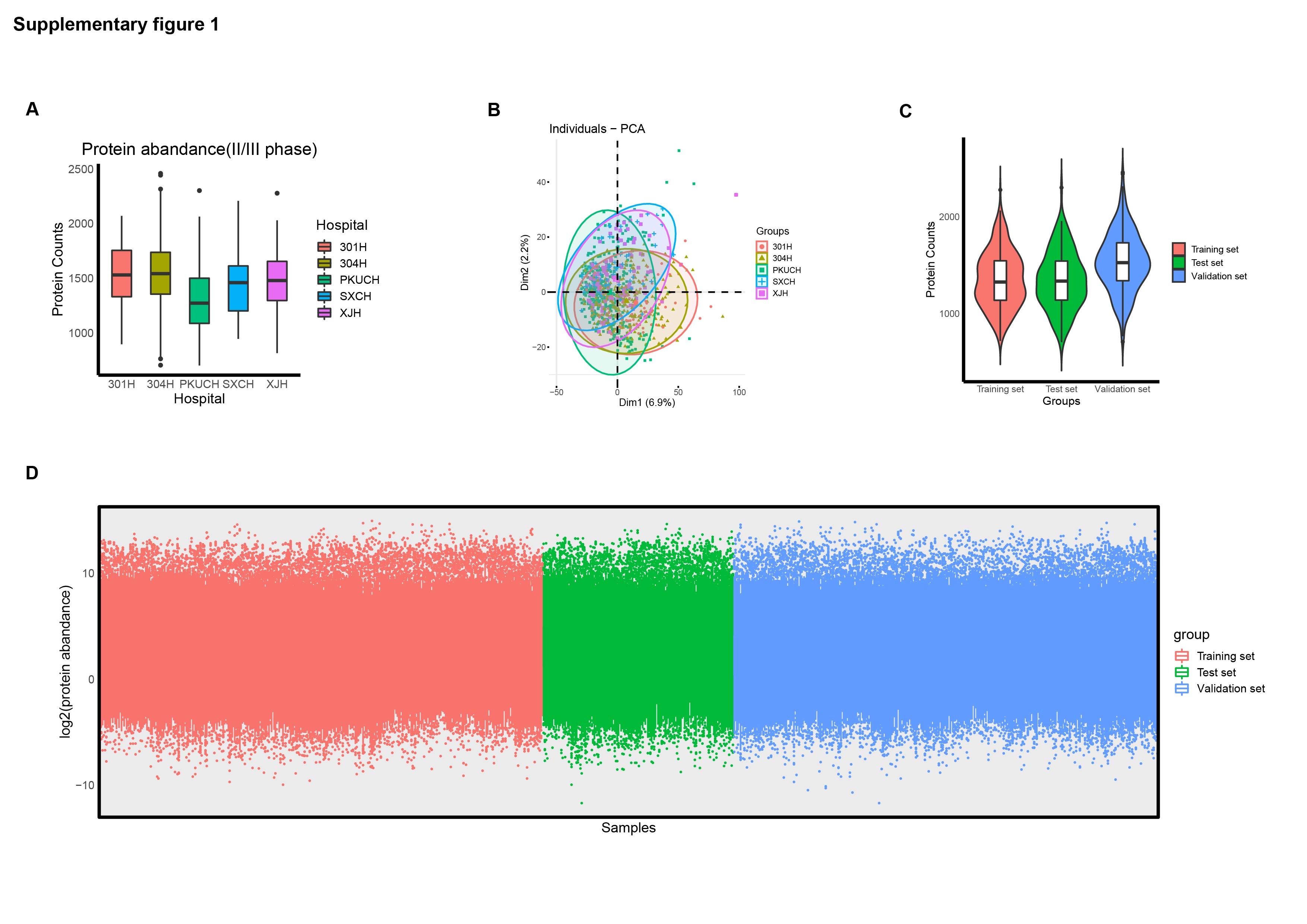

Supplement: Supplementary Figure 1 — Data quality evaluation. (A) Box plot of protein identification numbers in five hospitals. (B) PCA of 833 samples in five hospitals. (C) Violin plot of protein identification numbers in three datasets. (D) Box plot of protein abundance in each sample in three datasets. [file Image_1.jpeg]

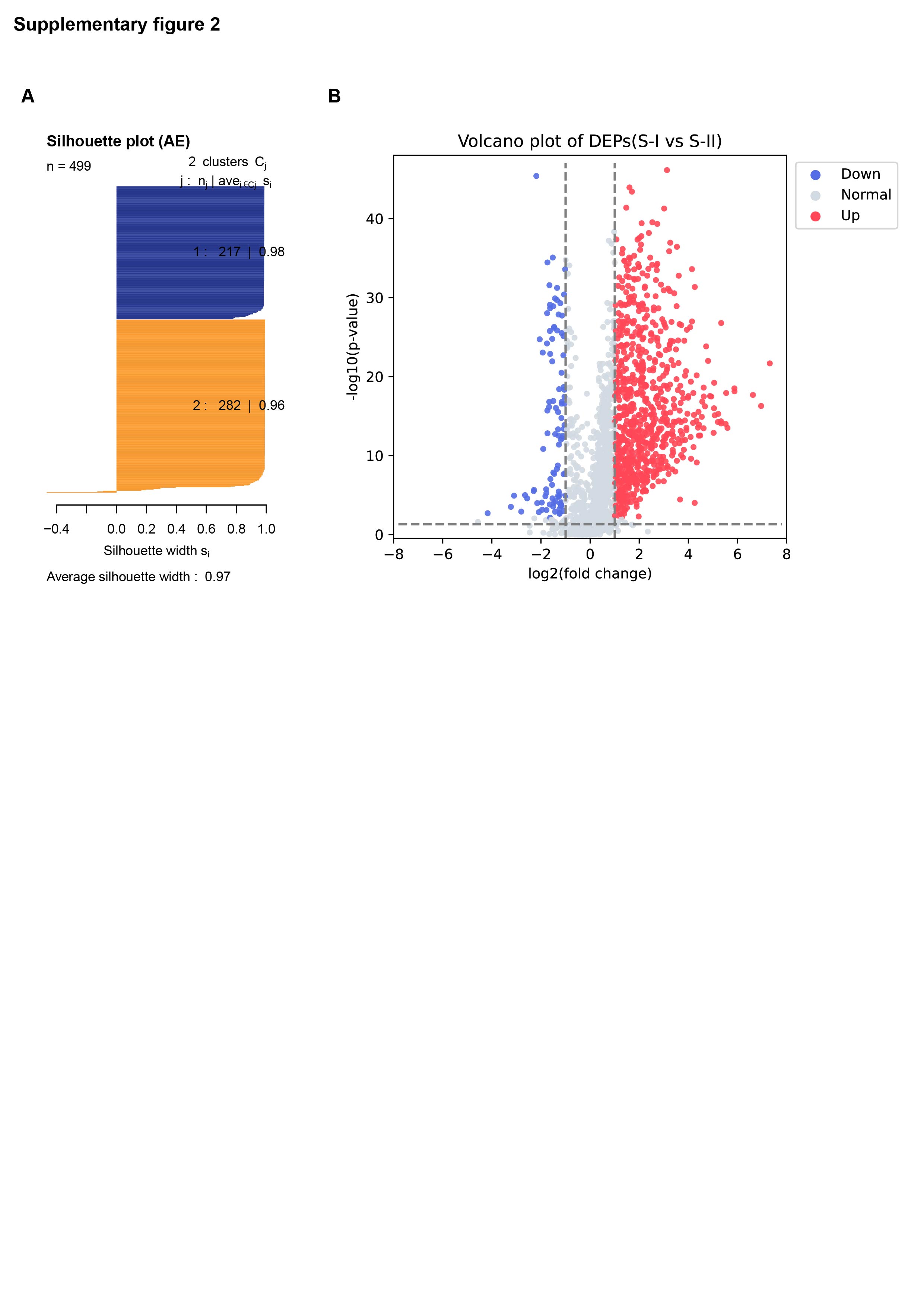

Supplement: Supplementary Figure 2 — The clustering result and differentially expressed proteins of the molecular subtypes in discovery set. (A) The silhouette plot of clustering results obtained from AE. (B) Volcano plot of differentially expressed proteins in two subtypes. [file Image_2.jpeg]

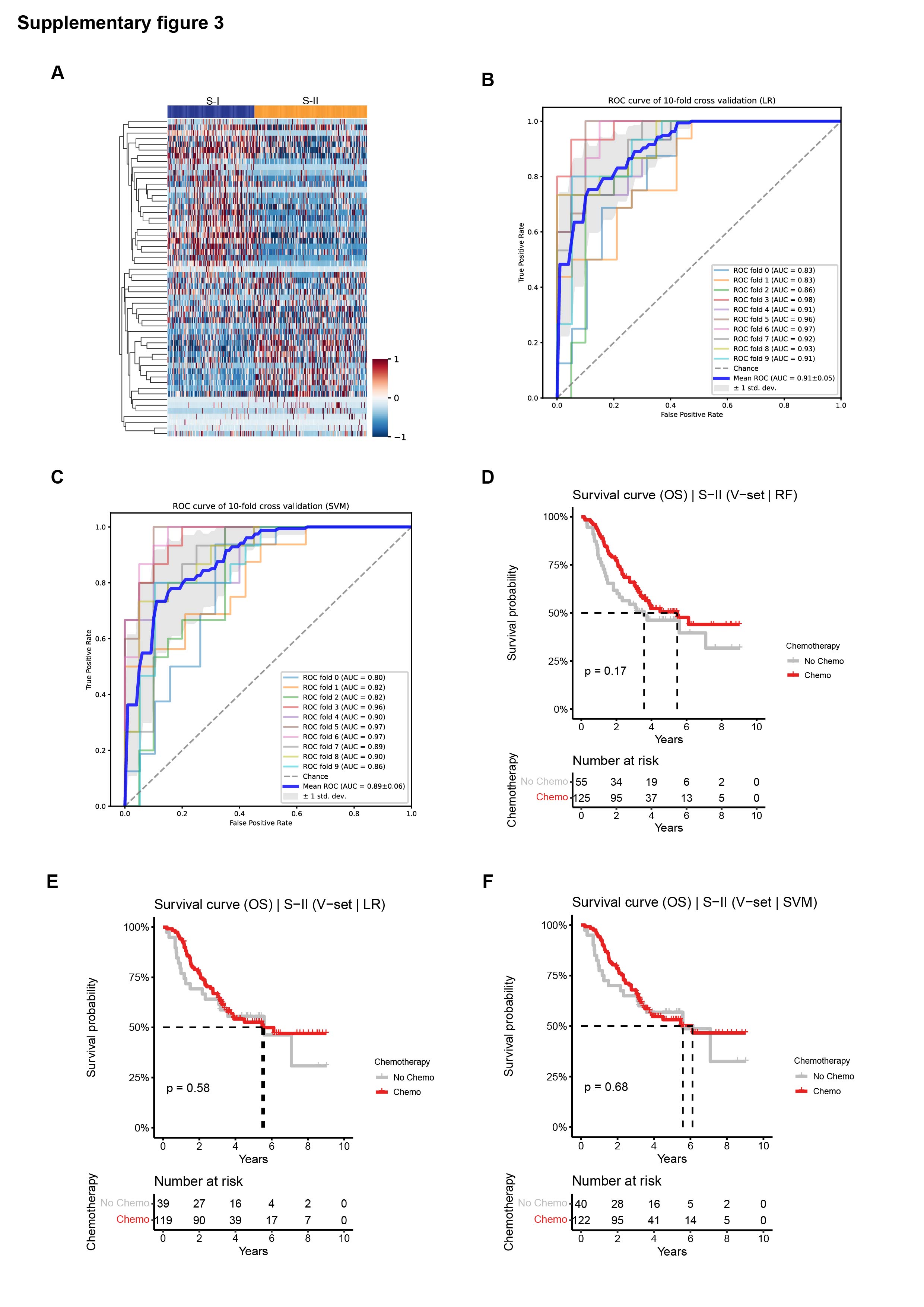

Supplement: Supplementary Figure 3 — Classifiers were established to predict survival-risk labels for samples in independent set. (A) A heat map based on the 56 selected proteins expression. (B, C) The ROC curve of LR and SVM on training set with 10-fold CV, respectively. (D–F) The OS by chemotherapy status for S-II predict by RF, LR, and SVM, respectively. [file Image_3.jpeg]

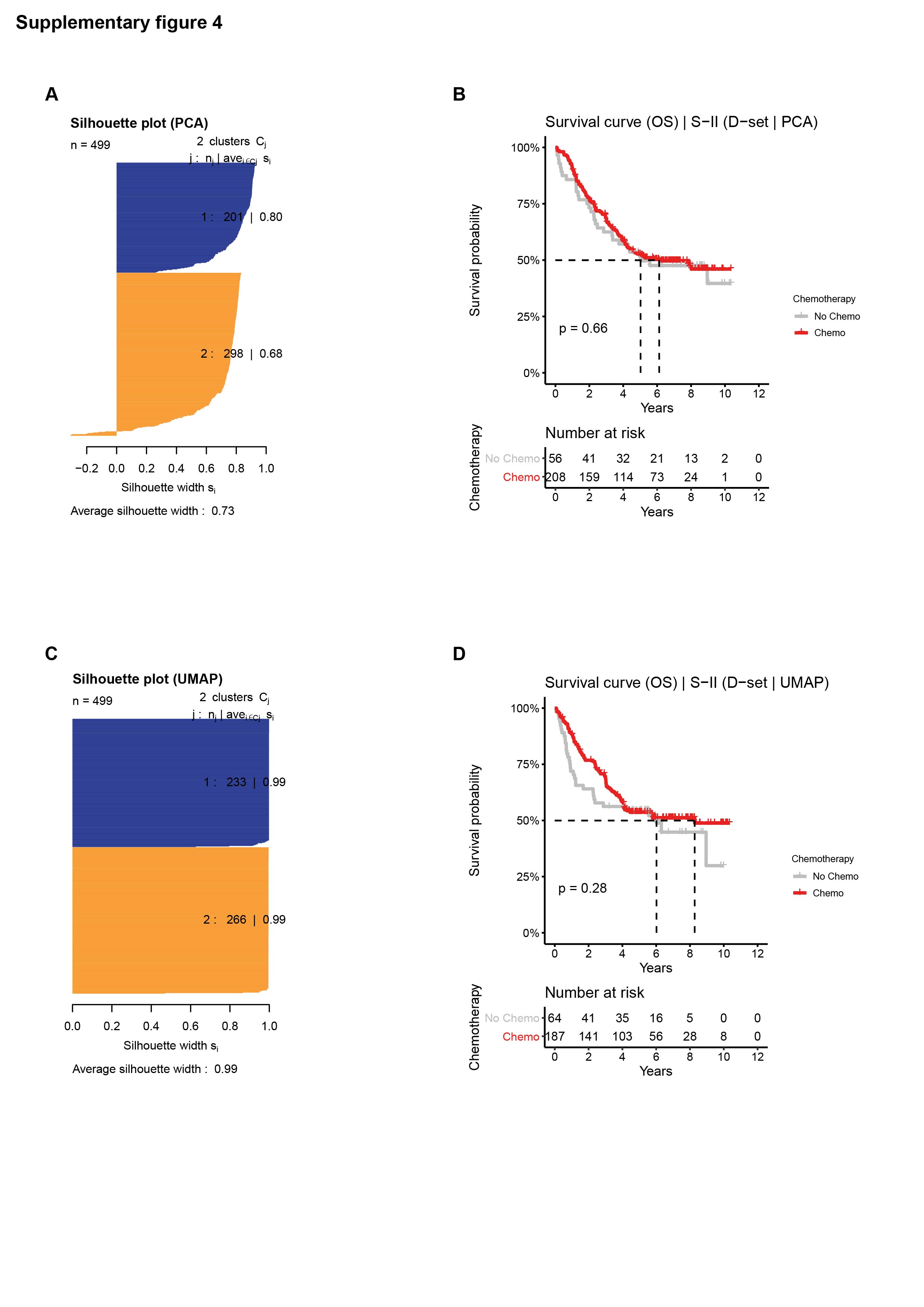

Supplement: Supplementary Figure 4 — The clustering results obtained from two alternative approaches in discovery set. (A) The silhouette plot of clustering results and (B) the OS by chemotherapy status for S-II obtained from PCA. (C) The silhouette plot of clustering results and (D) the OS by chemotherapy status for S-II obtained from UMAP. [file Image_4.jpeg]

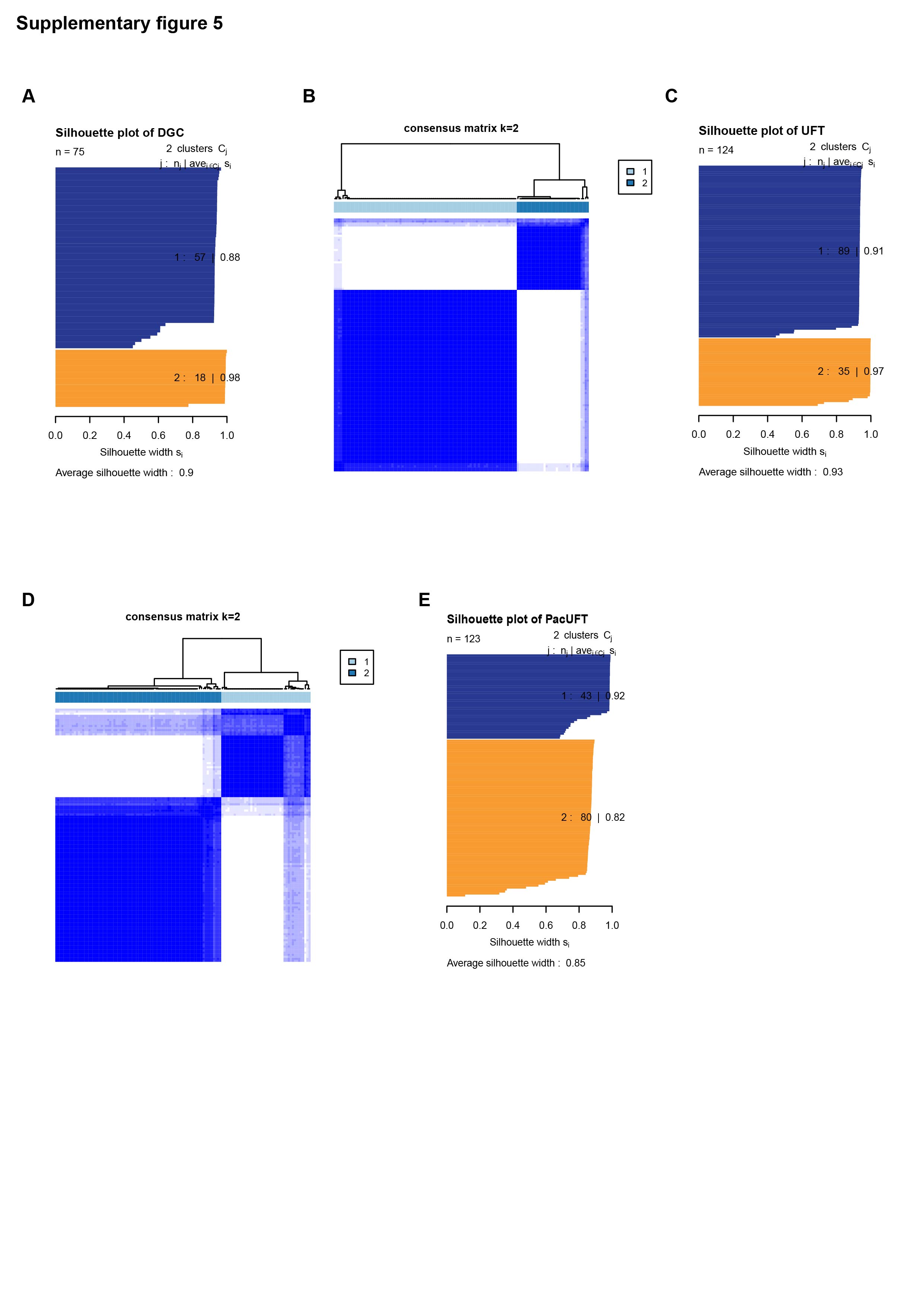

Supplement: Supplementary Figure 5 — The clustering results obtained by the protein features transformed from AE in the external validation set. (A) The silhouette plot of clustering results in DGC. (B, C) The clustering results for UFT-treated and (D, E) PacUFT-treated patients in the external validation set. [file Image_5.jpeg]
